# Intralobular pulmonary sequestration of the right upper lobe supplied by ascending aorta

**DOI:** 10.1093/icvts/ivad124

**Published:** 2023-07-31

**Authors:** Xiao-Xia Wang, Hong-Sheng Zhang, Yun-Xia Sui, Jie Liu

**Affiliations:** Department of Clinical Oncology, Weihai Central Hospital, The Affiliated Hospital of Qingdao University, Shandong, China; Department of Radiology, Weihai Central Hospital, The Affiliated Hospital of Qingdao University, Shandong, China; Department of Clinical Oncology, Weihai Central Hospital, The Affiliated Hospital of Qingdao University, Shandong, China; Department of Radiology, Weihai Central Hospital, The Affiliated Hospital of Qingdao University, Shandong, China

**Keywords:** pulmonary sequestration, right upper lobe, ascending aorta

## Abstract

Pulmonary sequestration (PS) is a rare congenital lung malformation that is more common in the left lower lobe. In 95% of cases, the artery supplying the sequestration usually originates from the thoracic and abdominal aorta. We report a rare intralobular PS case for a feeding artery from the ascending aorta. Angio-computed tomography should be performed for diagnosis once PS is suspected.

## INTRODUCTION

Pulmonary sequestration (PS) is a rare congenital lung malformation that is more common in the left lower lobe. In 95% of cases, the artery supplying the sequestration usually originates from the thoracic and abdominal aorta. We report a rare intralobular PS case for a feeding artery from the ascending aorta. Angio-computed tomography (CT) should be performed for diagnosis once PS is suspected.

## CASE PRESENTATION

A female aged 50–60 years was admitted to our Department of Clinical Oncology because of abnormalities detected by chest X-ray. Chest X-ray revealed a mass in the right upper lobe. She came to our hospital for surgery because neoplastic lesions could not be excluded. There was no evidence of tumour by bronchoscopy and PET–CT. Blood tests showed that tumour markers were negative for CEA, CA125, SCC and NSE.

**Figure 1:**
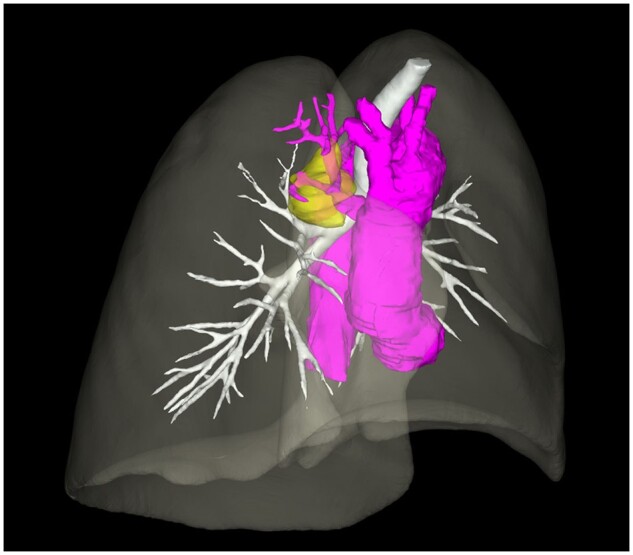
Three-dimensional reconstruction of the thorax shows the soft tissue mass (yellow), and the abnormal branching to sequestration arising from the ascending aorta (purple).

**Figure 2:**
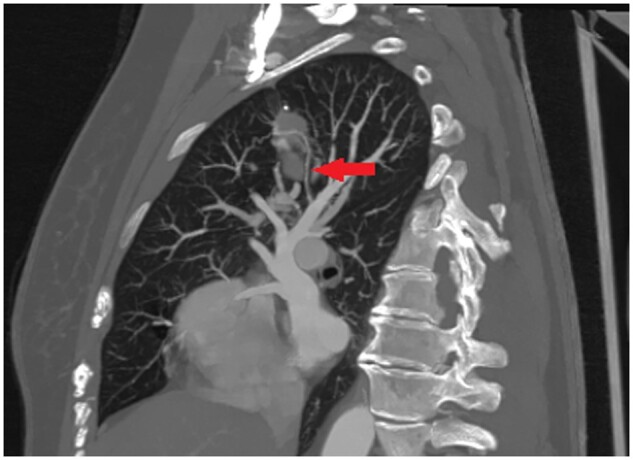
The venous drainage was mainly via the pulmonary veins.

The examination for tubercle bacillus was negative. CT scan showed a cyst in the upper lobe of the right lung. CT three-dimensional reconstruction of the thorax shows the soft tissue mass (yellow), and the abnormal branching to sequestration arising from the ascending aorta (purple) (Fig. 1). The venous drainage was mainly via the pulmonary veins (Fig. 2). The patient declined surgical treatment due to the benign lesion. The patient was doing well at a 2-year follow-up.

## DISCUSSION

PS constitutes ∼0.15–6.4% of all congenital lung malformations [1]. Pneumonia is a common symptom and haemoptysis may also occur. The most common location of PS is the left lower lobe [2]. The thoracic and abdominal aortas are the most common arterial supply. PS is often misdiagnosed as lung cancer, pulmonary cysts, mediastinal tumours, lung infection and bronchiectasis. CT can not only confirm the correction between the sequestered lung and arterial supply but also clearly display the number, origin and direction of abnormal supplying arteries through 3D VR technology. Conservative treatment is an option in asymptomatic patients. It has been firmly established that symptomatic patients require treatment [3]. The treatment of choice is surgical ligation of the anomalous vessel in combination with lung resection. Feeding arteries can also be severed through thoracoscopic surgery and vascular intervention. Angio-CT is an important examination technique for the diagnosis and preoperative assessment of PS.

## Data Availability

The data underlying this article will be shared on reasonable request to the corresponding author.
